# Application of Blind Deblurring Reconstruction Technique to SPECT Imaging

**DOI:** 10.1155/2007/63750

**Published:** 2007-12-06

**Authors:** Heng Li, Yibin Zheng

**Affiliations:** ^1^Department of Radiation Physics, MD Anderson Cancer Center, University of Texas, Houston, TX 77030, USA; ^2^Department of Electrical and Computer Engineering, University of Virginia, Charlottesville, VA 22904, USA

## Abstract

An SPECT image can be approximated as the convolution of the ground truth spatial radioactivity with the system point spread function (PSF). The PSF of an SPECT system is determined by the combined effect of several factors, including the gamma camera PSF, scattering, attenuation, and collimator response. It is hard to determine the SPECT system PSF analytically, although it may be measured experimentally. We formulated a blind deblurring reconstruction algorithm to estimate both the spatial radioactivity distribution and the system PSF from the set of blurred projection images. The algorithm imposes certain spatial-frequency domain constraints on the reconstruction volume and the PSF and does not otherwise assume knowledge of the PSF. The algorithm alternates between two iterative update sequences that correspond to the PSF and radioactivity estimations, respectively. In simulations and a small-animal study, the algorithm reduced image blurring and preserved the edges without introducing extra artifacts. The localized measurement shows that the reconstruction efficiency of SPECT images improved more than 50% compared to conventional expectation maximization (EM) reconstruction. In experimental studies, the contrast and quality of reconstruction was substantially improved with the blind deblurring reconstruction algorithm.

## 1. INTRODUCTION

Iterative methods are commonly used in single photon emission computed tomography (SPECT) reconstruction because of their ability to handle incomplete data and to incorporate a priori information in the process. Because the sensitivity and resolution of SPECT are complex functions of many factors, such as scattering, medium attenuation, and collimator response, it is difficult to incorporate these factors analytically into the reconstruction process. Some researchers have shown that for a parallel-hole camera [1], the point-spread function (PSF) of the gamma ray detector without scatter could be approximated by a Gaussian function, whereas the PSF with scattered photons can be described by convolving a zero-order Bessel function of the second kind with the PSF without scatter. The overall PSF of the gamma camera depends on the source location inside the object and the shape of the object; this PSF can then be incorporated into the iterative reconstruction algorithm [2]. However, because of the complexity and the object-dependent nature of the PSF model, it is impractical to apply the exact form of the PSF directly for pinhole imaging. In cone-beam geometry, the scatter caused by the imaged object is also hard to determine. However, the combined effect of scatter and detector PSF is approximately the same as low-pass filtering of the projection image. Therefore, image restoration and deconvolution techniques can be performed on either the projection image [3, 4] or the reconstruction image [5, 6]. Although the results of these techniques are often improving image quality, the PSF functions used in these techniques are either assumed to be known or are estimated by neglecting the physics of the SPECT system, and some extra artifacts might be introduced.

Inspired by the idea of blind deconvolution introduced by Holmes [7], we proposed a phenomenological model that incorporates the effects of attenuation, scatter, and detector response into the reconstruction process. The algorithm is an iterative expectation-maximization (EM) algorithm. We modified the photon transition probability matrix to account for attenuation and included a convolutional kernel in the forward projection operation to model scatter and detector response. We also proposed to use two iterative updates instead of one to reconstruct both the object and the PSF. The next section describes the development of the reconstruction algorithm.

## 2. BLIND DEBLURRING RECONSTRUCTION

### 2.1. Blind deblurring

Blind deblurring is a technique that permits recovery of an object from a set of “blurred” images in the presence of a poorly determined or unknown PSF, that is, deconvolving a signal without knowing the impulse response [8–10].

**Figure 1:**
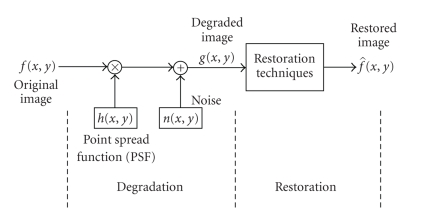
General process for image restoration.

Figure 1 shows the general process of image restoration from a degraded image. The measured image g (x, y) can be written as

(1) $$g(x,y)=f(x,y{)}^{*}h(x,y)+n(x,y)$$

with ∬h(x,y)dxdy=1 and ^*^ denotes convolution. In discrete form, (1) becomes

(2) $$g_{i}=\underset{j}{\sum }h_{i-j}f_{j}+n_{i}$$

with $\sum_{j}h_{j}=1$. Conventional linear and nonlinear deconvolution techniques require a known PSF. Assuming the PSF h is known, one can use the Richardson-Lucy deconvolution algorithm [11, 12] developed in the 1970s from Bayes's theorem [13] to perform the deconvolution. Assuming independent Poisson distribution for each pixel, the deconvolution process can be written as an iterative algorithm,

(3) $$f_{j}^{n+1}=f_{j}^{n}\underset{i}{\sum }\left(\frac{h_{i-j}g_{i}}{\sum_{k}h_{i-k}f_{k}^{n}}\right),$$

which intrinsically applies the positivity constraint and conserves energy.

However, the kernel h is often unknown in practice. Holmes [7] derived an iterative algorithm for finding the maximum likelihood solution for both the unblurred image and the PSF in the presence of Poisson statistics in the photon counting process [7]. The algorithm consists of two simultaneous Richardson-Lucy-like iterations:

(4) $$h_{j}^{n+1}=h_{j}^{n}\underset{i}{\sum }\left(\frac{f_{i-j}^{n}g_{i}}{\sum_{k}h_{i-k}^{n}f_{k}^{n}}\right),\;f_{j}^{n+1}=f_{j}^{n}\underset{i}{\sum }\left(\frac{h_{i-j}^{n+1}g_{i}}{\sum_{k}h_{i-k}^{n+1}f_{k}^{n}}\right).$$

Equation (4) is iterated until convergence occurs. The algorithm ensures strict positivity. However, this approach to blind deconvolution is likely to fail unless one can place very strong constraints on the properties of the PSF or the image. For example, research shows that if one applies the Holmes iteration to images from the Hubble space telescope with no constraints on the PSF, the unblurred image looks exactly like the data and the PSF is an impulse: a perfect fit to the data, but far from the truth. This is because the measured data are underdetermined and the number of unknown variables is too large. Constraints on the PSF are often adopted to better describe the problem [7], 0:f and h are positive and real and have finite support, and h is band limited. By applying these constraints, the number of variables is effectively reduced and images with improved quality can be obtained.

### 2.2. Blind deblurring reconstruction

Assuming Θ to be the voxel set and Ψ to be the detector pixel set, the EM algorithm [14] with attenuation correction for SPECT can be written as

(5) Fi(λ^n)=λ^in+1=λ^inHi∑jNjpije-〈l,μ〉j∑i′λ^i′npije-〈l,μ〉j,Hi=∑jpije-〈l,μ〉j=∑jhij,

where i∈Θ is in the voxel domain and j∈Ψ is in the detector domain. Because the main effect of scatter and detector PSF is the loss of resolution in the reconstructed image, or the broadening of a point source, one could model this effect as a convolution of the true radioactivity with a kernel $g_{i}$:

(6) fi=Poisson{λig*i},

where $f_{i}$ is the reconstructed radioactivity without scatter and PSF correction and ^*^ denotes two-dimensional linear convolution. Assuming $g_{i}$ can be estimated, one could first compute $f_{i}$ using standard EM iterations (5) (with ${\hat{\lambda }}_{i}$ being replaced by ${\hat{f}}_{i}$) and then deconvolve ${\hat{f}}_{i}$ with $g_{i}$. However, the ${\hat{\lambda }}_{i}$ so obtained is not the maximum likelihood estimate of $\lambda_{i}$ given $N_{j}$; also, the kernel $g_{i}$, which is the combined effect of scattering, the pinhole geometry, and the detector PSF, is generally a complex unknown function. Our experiences with such an approach indicate that the deconvolution step creates unwanted artifacts and noise. The new approach integrates deconvolution into the iterative reconstruction process.

Suppose the total number of emitted photons is Λ, the total number of detected photons is H, Λ≥H, $I_{k}$, k=1⋯Λ, $I_{k}\in \Theta$ denotes the location from which the kth photon was emitted under the ideal conditions in which no blurring is present, and $J_{q}$, q=1⋯H, J∈Ψ denotes the location where the qth photon is detected. We call these emission locations true emission points [7]. A finite number of these points form an inhomogeneous Poisson random-point process having the intensity function $\lambda_{i}$. Under ideal conditions, the number of detected photons at detector j is related to $I_{k}$ as

(7) $$P(J_{k}=j|I_{k}=i)=p_{ij}^{0},\;\;\;N_{j}=\overset{\Lambda }{\underset{k=1}{\sum }}I_{k}p_{ij}^{0},$$

Again, $p_{ij}^{0}$ denotes the probability that each photon emitted from position i will reach detector j. Under ideal conditions, Λ= H and $\sum_{j}p_{ij}^{0}=1$, meaning that each emitted photon will be detected by some detector unit. However, because of the presence of attenuation, a portion of emitted photons is lost. With that in mind, we use the following equation to denote the effective probability matrix:

(8) $$p_{ij}=p_{ij}^{0}e^{-{\langle l,\mu \rangle }_{j}}.$$

When blurring due to the combined effect of scattering, pinhole geometry, and detector PSF occurs, the positional measurement of each emission point is corrupted by a random translation. Let $Y_{k}$ denote this error vector, and then the measured data for detector pixel j is related to $I_{k}$ and $Y_{k}$ by

(9) $$P(J_{q}=j|I_{k}+Y_{k}=i)=p_{ij},$$

(10) $$N_{j}=\overset{\Lambda }{\underset{k=1}{\sum }}(I_{k}+Y_{k})p_{(I_{k}+Y_{k})j},$$

where $Y_{k}$ is statistically independent of all $I_{k}$'s, and they are all statistically independent of each other for all photons emitted and identically distributed with a probability density $\gamma_{i}=\Lambda g_{i}$, indicating the presence of a blurring kernel. It should be noted that Λ> H and $\sum_{j}p_{ij}<1$. It can also be shown [7] that this set of error vectors constitutes an inhomogeneous Poisson random-point process. Therefore, using the Laplace transform of (10), the expectation of the detected number of photons can be evaluated as

(11) E{Nj}=∑i(λig*i)pij=∑i∑i′λi′gi−i′pij.

On the other hand,

(12) $$\begin{array}{ll} E\{N_{j}\} & =E\{\underset{i}{\sum }N_{i}P(J_{q}=j|I_{k}=i)\}\\ & =\underset{i}{\sum }E\{N_{i}\}P(J_{q}=j|I_{k}=i)\\ & =\underset{i}{\sum }\lambda_{i}P(J_{q}=j|I_{k}=i). \end{array}$$

From (11) and (12), noticing the independence of $Y_{k}$ and $I_{k}$, we have

(13) $$P(J_{q}=j|I_{k}=i)=\underset{i^{\prime }}{\sum }g_{i-i^{\prime }}p_{i^{\prime }j}.$$

Similarly,

(14) $$P(J_{q}=j|Y_{k}=i)=\frac{1}{\Lambda }\underset{i^{\prime }}{\sum }\lambda_{i-i^{\prime }}p_{i^{\prime }j}.$$

If $N_{i}$ is the actual number of photons emitted from position i and $B_{i}$ is the number of photons having error vectors within voxel i, then they follow the Poisson distribution with mean $\lambda_{i}$ and $\gamma_{i}$, respectively. In our application, $N_{j}$, H, and $J_{q}$ are known measured data and $N_{i}$ and $B_{i}$ or $I_{k}$ and $Y_{k}$ are two sets of substantially identical unknown data. We can now construct the EM algorithm, which consists of two steps: the expectation (E) step and the maximization (M) step. In the E step, complete data, that is, $N_{i}$ and $B_{i}$ or $I_{k}$ and $Y_{k}$, are formed using expectations of the missing data. Here the set of true emission vectors, I, and the set of error vectors, Y, are noted as

(15) $$\begin{array}{ll} I & =\{I_{1},I_{2},I_{3}...\},\\ Y & =\{Y_{1},Y_{2},Y_{3}...\}. \end{array}$$

Then the log likelihood of I can be expressed as

(16) $$l(I|\text{λ})=-\underset{i}{\sum }\lambda_{i}+\underset{i}{\sum }\text{ln}(\lambda_{i})N_{i},$$

where λ is the vector notation for all $\lambda_{i}$. Similarly, the log likelihood of B can be written as

(17) $$l(Y|g,\Lambda )=-\underset{i}{\sum }\gamma_{i}+\underset{i}{\sum }\text{ln}(\gamma_{i})B_{i},$$

where g is the vector notation for all $g_{i}$. The log likelihood of the complete data then can be equivalently expressed in two ways, assuming I or Y is known, that is,

(18) $$\begin{array}{ll} l(I,Y|\text{λ},g,\Lambda ) & =l(I|\text{λ},g,\Lambda )+l(Y|\lambda ,g,\Lambda )\\ & =-\underset{i}{\sum }\lambda_{i}+\underset{i}{\sum }\text{ln}(\lambda_{i})N_{i}+\overset{\Lambda }{\underset{k=1}{\sum }}\text{ln}(g(Y_{k}))\\ & =-\underset{i}{\sum }\gamma_{i}+\underset{i}{\sum }\text{ln}(\gamma_{i})B_{i}+\overset{\Lambda }{\underset{k=1}{\sum }}\text{ln}(\lambda (I_{k})/\Lambda ). \end{array}$$

Let $C^{n}$ denote the condition, that is, the collected data $N_{j}$, and the estimation of $\lambda_{i}^{n}$, $g_{i}^{n}$, and $\Lambda^{n}$, then we can write the expectation step in the following form:

(19) $$\begin{array}{ll} E\{l(I,Y|C^{n})\} & =-\underset{i}{\sum }\lambda_{i}+\underset{i}{\sum }\text{ln}(\lambda_{i})E\{N_{i}|C^{n}\}\\ & \;+\overset{\Lambda }{\underset{k=1}{\sum }}E\{\text{ln}(g(B_{k}))|C^{n}\},\\ E\{l(I,Y|C^{n})\} & =-\underset{i}{\sum }\gamma_{i}+\underset{i}{\sum }\text{ln}(\gamma_{i})E\{B_{i}|C^{n}\}\\ & \;+\overset{\Lambda }{\underset{k=1}{\sum }}E\{\text{ln}(\lambda (I_{k})/\Lambda )|C^{n}\}, \end{array}$$

where by definition,

(20) $$\begin{array}{ll} E\{N_{i}|C^{n}\} & =\underset{j}{\sum }N_{j}P(I_{k}=i|J_{q}=j),\\ E\{B_{i}|C^{n}\} & =\underset{j}{\sum }N_{j}P(Y_{k}=i|J_{q}=j), \end{array}$$

and using Bayes's theorem,

(21) $$P(A|B)=\frac{P(A)P(B|A)}{\sum_{A}P(A)P(B|A)}.$$

We have

(22) P(Ik=i∣Jq=j)=P(Ik=i)P(Jq=j∣Ik=i)∑k′=1Λ^nP(Jq=j∣Ik′=i)P(Ik′=i).

Noticing $P(I_{k}=i)={\hat{\lambda }}_{i}^{n}/{\hat{\Lambda }}^{n}$ and using (13), we now have

(23) P(Ik=i∣Jq=j)=λ^in∑i′′′gi′−i′′′pi′′′j∑i′λ^i′n∑i′′′gi′−i′′pi′′j.

Thus,

(24) E{Ni∣Cn}=∑jNjλin∑i′′′gi−i′′′pi′′′j∑i′λi′n∑i′′gi′−i′′pi′′j=λ^in∑jNj∑i′′gi−i′′pi′′j∑i′λ^i′n∑i′′gi′−i′′pi′′j=λ^in∑jNj∑i′′gi−i′′pi′′j∑i′(λ^i′n*gi′)pi′j.

Similarly, we have

(25) E{Bi∣Cn}=g^in∑jNj∑i′′λi-i′′pi′′j∑i′(λi′*g^i′n)pi′j.

For the M step, (19) is maximized simultaneously. The following update equation maximize the two log likelihoods:

(26) λ^n+1=E{Ni∣Cn},g^n+1=E{Bi∣Cn}Λ^n,Λ^n+1=∑iλ^in+1.

In summary, the following iteration converges to the maximum likelihood estimate of $\lambda_{i}$:

(27) λ^in+1=λ^in∑jNj∑i′′gi−i′′pi′′j∑i′(λ^i′ng*i′)pi′j,

(28) g^in+1=g^inΛ^n∑jNj∑i′′λi-i′′pi′′j∑i′(λi′g^*i′n)pi′j,

(29) Λ^n+1=∑iλ^in+1.

The initial $\lambda_{i}^{0}$ is an image of all 1's, $g_{i}^{0}$ is the same image normalized to 1, and $\Lambda^{0}$ is the total number of detected photons. Equations (27) and (28) are then evaluated to acquire a new pair of estimates of λ and g. The PSF of the SPECT system is assumed to be real, nonnegative, and band limited. Letting $F_{z}$ be the frequency components of the PSF that are known to be zero, the band-limited constraints are incorporated by executing the following steps in each iteration:

1. the Fourier transform of ${\hat{g}}^{n+1}$ is taken, and any frequency components that lie within $F_{z}$ are set to zeros;
2. the inverse Fourier transform of (1) is taken, and any negative or complex values in the spatial domain are set to zeros.

The first step of the process ensures the band-limited constraint, and the second step ensures the reality and nonnegativity of the PSF. Realness and nonnegativity are implicitly applied to λ. Equations (27), (28), and (29) and steps (1) and (2) are then iterated until convergence occurs.

The blind deblurring reconstruction algorithm estimates both the spatial radioactivity distribution and the system PSF from the set of blurred projection images. The iteration for reconstruction can be understood as replacing the forward projector in the original EM (denominator of (5)) with the new projector using the convolved radioactivity map, and the iteration for solving the PSF can be understood as blind deblurring. This iteration differs from the general image blind deconvolution in the sense that the kennel is partly known; $p_{ij}$, the known transfer matrix, is in fact part of the blurring kennel. In addition, instead of deconvolving an image where both the input and output are two-dimensional images, the input of blind deblurring reconstruction is a series of projection images, and the output is a three-dimensional image array.

## 3. METHODS

**Figure 2:**
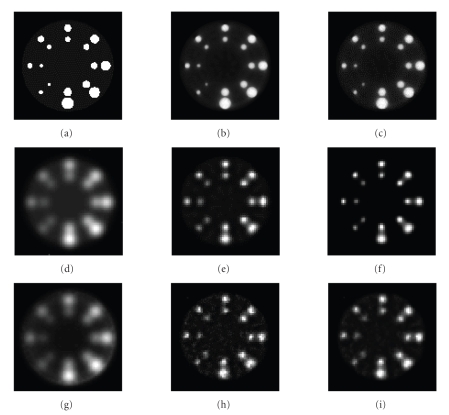
Reconstruction results for the ring of spheres phantom. (a) The ring of spheres phantom; (b) EM reconstruction of ideal projection data; (c) the same ring of spheres phantom with Poisson distribution with total counts of 10^6^; (d) EM reconstruction with no PSF correction from blurry projection data of (a); (e) blind deblurring reconstruction; (f) reconstruction with known PSF correction; (g) EM reconstruction with no PSF correction from blurry projection data, with total photon count in projection data 10^6^; (h) blind deblurring reconstruction; (i) reconstruction with known PSF correction.

We used both simulation and experimental data to validate and evaluate the performance of the blind deblurring reconstruction. For computer simulations, we used the ring of spheres phantom shown in Figure 2(a). The phantom consisted of 16 spheres on the same x-z plane with diameters from 1 mm to 6 mm (1, 1.5, 2, 2.3, 2.6, 2.7, 3.1, 3.3, 3.4, 3.8, 4.2, 4.3, 4.8, 5.4, and 6 mm), and all of the spheres had the same magnitude, 1.1. The background was a big sphere with a 28 mm radius and a magnitude of 0.1. Pinhole geometry simulating a physical small-animal imaging system [15], which will be described in more detail below, was adopted in the study. The simulated pinhole had an effective diameter of 1 mm and an acceptance angle of 100 degrees. The simulated detector had 60 × 60 pixels with a pixel size of 2 mm and was placed 71 mm from the pinhole. The radius of rotation (ROR) of the simulation was 4 cm, and the magnification factor was 1.7. The radius of the reconstructed field of view was 3 cm. The blurring kernel, $g_{i}$, was simulated using a point source reconstruction from the physical system.

We then conducted phantom studies on the pinhole SPECT imaging system with a gamma detector assembled at the Thomas Jefferson national accelerator facility. The detector consists of a 2 × 2 array of Hamamatsu H8500 position-sensitive photon-multiplier tubes coupled to a 1.3 × 1.3 × 6 mm pixilated NaI(Tl) crystal array with 1.6 mm center-to-center spacing, providing about 80% absorption efficiency at 140 keV. The intrinsic detector full width half maximum (FWHM) of the detector is 1.8 mm. The pinhole, fabricated by Mikro Systems, Inc., Charlottesville, VA, USA, was composed of a tungsten-polymer composite (with a linear attenuation coefficient of 2.1 mm^-1^). The pinhole's diameter was 1 mm, and the acceptance angle was 100 degrees. Specified details of the phantoms being imaged are presented in Section 4.

We also performed the reconstruction technique in an animal study, in which we imaged cardiac inflammation in a mouse resulting from ischemia caused by the injection of Tc 99m-labeled antibody.

## 4. RESULTS

We analyzed the reconstruction results from the same projection data, either computer generated or experimentally collected, using different iterative reconstruction techniques. Attenuation and attenuation corrections were included in all of the simulations and experiments unless specified otherwise. Also, all reconstruction images displayed and analyzed are the results after sufficient numbers of iterations and convergence were achieved.

### 4.1. Ring of spheres phantom study

**Figure 3:**
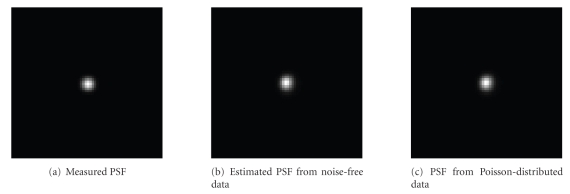
Estimated point spread function (PSF) for blind deblurring reconstruction.

The blind deblurring reconstruction approach described in Section 3 was first validated using a simulated phantom, the ring of spheres phantom shown in Figure 2(a), with and without the Poisson distribution model. Figure 2(b) is the EM reconstruction of the projections from ideal projection data of Figures 2(a) and 2(c) is the EM reconstruction from nonblurry projection data following Poisson distribution with a total count of 10^6^. Projections were generated from Figure 2(a) with the PSF kernel of a physically measured point source (Figure 3(a)) and was then reconstructed using different techniques, as demonstrated in Figures 2(d)–2(f). Projections with the same PSF and following Poisson distribution with total count of 10^6^ were also generated and the reconstructions are shown in Figures 2(g)–2(i).

Figures 2(d) and 2(g) are reconstructions with no PSF correction, and the images are blurry, as expected. Figures 2(e) and 2(h) demonstrate the result of blind deblurring reconstruction, whereas Figures 2(f) and 2(i) are the reconstructions with known PSF. By comparing the reconstructions with and without correction, one can observe that with the blind deblurring method, both the resolution and contrast of the reconstruction image are considerably improved, and the blind deblurring technique can achieve a quality similar to that as achieved with known PSF correction.

Figure 3(a) shows the measured PSF of the small animal pinhole SPECT imaging system [15] using a single shot of point source placed precisely at the isocenter. The FWHM of the measured PSF on detector was 1.6 mm. This PSF, which is geometry dependent, was assumed space invariant for all voxels and used to generate simulated projection data. The estimated PSF from blind deblurring EM reconstruction (projected onto detector) is shown in Figures 3(b) and 3(c). The correlation between the estimated PSF and the real PSF was 96% and 94% for noise-free and Poisson-distributed blind deblurring reconstructions, respectively.

**Figure 4:**
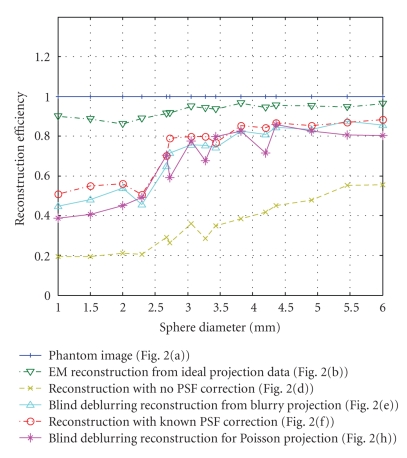
Reconstruction efficiency for spheres with different diameters.

Figure 4 shows the reconstruction efficiency for different reconstruction methods, which indicates the effectiveness of the reconstruction. The voxel values of each sphere were summed and averaged. The diameters of the spheres varied from 1 mm to 6 mm, or about 2 to 10 pixels on the detector grid. The object-to-background ratio was 11:1 for all spheres. The plot shows that the EM algorithm was biased even for the ideal case, mainly because of the pixelization or partial volume effect. Using the blind deblurring technique, the reconstruction mass can be recovered close to the ideal reconstruction. For small objects (diameter <2 mm in this study), the reconstructed object intensities were only less than 60%, even with PSF correction. For objects with greater diameters, the efficiency could be recovered to more than 80%. The efficiency of the blind deblurring reconstructions was about the same for noise-free and Poisson-distributed cases, and is improved by more than 50% over EM reconstruction. Our results also indicated that the reconstruction efficiency using the blind deblurring reconstruction was comparable to the efficiency using a known PSF correction.

### 4.2. Jaszczak phantom study

**Figure 5:**
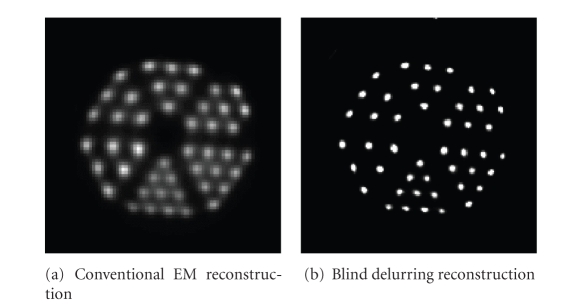
Slice from Jaszczak phantom reconstruction.

**Figure 6:**
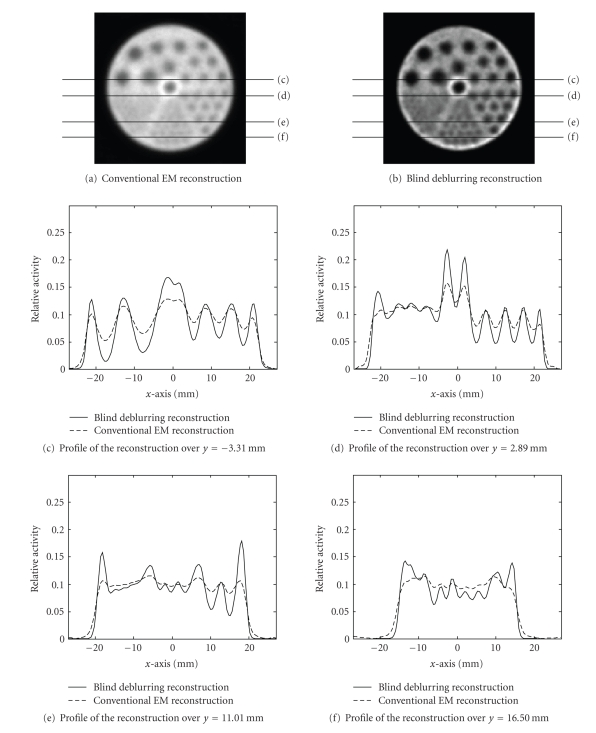
Slice from cold-rod phantom reconstruction. Four sets of line profiles were drawn in the same slice.

A hot-rod and a cold-rod Jaszczak phantom were imaged using the small-animal imaging system [15], and the reconstructions are shown in Figures 5 and 6. Figure 5 shows reconstructions of a slice from the first Jaszczak phantom with 1 million photon counts. The phantom was a hollow acrylic cylinder with an outer diameter of 30 mm. The phantom has six sections of rods with diameter ranged from 1.2 to 1.7 mm, each section has 6–10 rods drilled along the longitudinal axis, with center-to-center spacing of twice the rod diameter. In this study, the rods were filled with 0.8 mCi of technetium Tc 99m-solution, and 120 evenly spaced projections were taken over 360 degrees at 15 seconds per projection, and the total acquisition time was 37 minutes. The ROR was 3.1 cm, and the magnification factor was about 3.

Figure 5(a) shows the reconstruction using conventional EM with no correction for blurring, and Figure 5(b) shows the reconstruction using the blind-deblurring technique. Both reconstruction images have pixel sizes of 0.37 mm and slice thicknesses of 1.6 mm. The smallest set of rods with diameter of 1.2 mm is not resolved well in conventional EM, while shows up sharp and clearly in blind deblurring EM reconstruction. The mean FWHM measurements for different sets of rods are listed in Table 1. 

The error for FWHM measurements in conventional EM reconstruction is up to 15% and reduced to within 5% for blind deblurring reconstruction.

Figure 6 shows the reconstruction of the cold-rod phantom. The phantom had an inner diameter of 4.5 cm and consisted of six sets of rods with diameters ranging from 1.2 to 4.8 mm. In this study, 10 mCi Tc 99m-labeled radionuclide was distributed in the phantom, 120 evenly spaced projections were taken over 360 degrees at 2 minutes per projection, the total acquisition time was 4 hours, and the total photon count was 11 million. The ROR was 70 mm, and the magnification factor was 1.57. Again, the image quality and contrast were greatly improved, as shown in the images. The line profiles indicate contrast improvement for rods with diameters of 1.6 mm (second smallest) and larger. The uniformity of radionuclide distribution in the phantom was well preserved using the blind deblurring reconstruction technique.

### 4.3. Small-animal study

**Figure 7:**
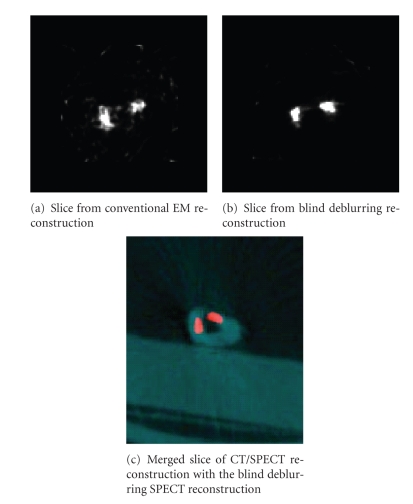
One slice of a mouse-heart reconstruction.

We also used the blind deblurring reconstruction technique in a study of cardiac inflammation (i.e., a heart attack) in a mouse resulting from ischemia caused by injection of Tc 99m-labeled antibody. How the antibody accumulated in the heart was of great interest. Approximately 900 μCi of Tc 99m-labeled antibody was injected into a mouse. The mouse was euthanized 6 hours after the injection, and the heart, which had a diameter of less than 1 cm, was removed and scanned on a CT/SPECT dual-modality scanner. CT projection data were acquired at 1 second per frame for a total scan time of 6 minutes, and SPECT projection data were acquired at 60 seconds per frame for a total scan time of approximately 1 hour. Figure 7 shows a reconstruction slice from the SPECT reconstruction and a fused slice of CT and SPECT reconstruction. No attenuation correction was made in the reconstruction process for this study.

As apparent in a comparison of Figures 7(a) and 7(b), the blind deblurring reconstruction resulted in better localization of radioactivity and higher contrast than the conventional EM reconstruction did, and the merged CT/SPECT registration Figure 7(c) shows a promising image.

## 5. DISCUSSIONS AND CONCLUSION

As demonstrated in both computer simulation and physical experiments, our new blind deblurring reconstruction technique substantially improved the quality and contrast of the reconstruction. This algorithm not only reconstructed the radiotracer map but also determined the complex PSF of the system. The masses and edges were well preserved in the reconstruction image, a feature that can be extremely useful when physicians need to localize or tally the activities in a possible tumor. However, some issues needed to be addressed in the reconstruction image; as seen in Figure 6(b), there was some degree of overshoot on the edge of the phantom in the reconstruction, which might be due to the nature of maximum likelihood estimation, as discussed by Snyder et al. in [16], and worth investigating. Further studies will also include how the level of distortion affects the performance of the algorithm and the performance of the algorithm applied to different organs.

## Figures and Tables

**Table 1:** Mean FWHM measurements for different groups of cylinders.

Real diameter (mm)	1.20	1.30	1.40	1.50	1.60	1.70
Mean FWHM in conventional EM reconstruction (mm)	1.42	1.53	1.61	1.70	1.84	1.93

Mean FWHM in blind deblurring EM reconstruction (mm)	1.14	1.33	1.38	1.47	1.61	1.69
